# Meetings of Societies

**Published:** 1931

**Authors:** 


### Meetings of Societies

### Bristol Medico-Chirurgical Society

The seventh meeting of the session was held at the University on 8th April, 1931. The President was in the Chair.

Dr. G. B. BUSH showed and described X-ray films, Dr. A. D. FRASER pathological specimens, and Mr. GRIFFITHS and Dr. ROGERS a microscopic specimen of a sarcoma arising from the stomach.

Dr. A. L. TAYLOR then gave a paper on some "**Intestinal Abnormalities and their Clinical Significance,**" illustrated by lantern slides and specimens. This paper appears in the current issue.

In the discussion that followed the President inquired whether diverticula of the Fallopian tube were of the nature of heterotopias, and whether adeno-myomata of the uterus containing endometrial tissue were of the same class.

Dr. ROGERS congratulated Dr. Taylor on his work on the heterotopias. He remarked that while transplants in the anterior gastric wall took well, with no ulceration, ulceration occurred if the site were the lesser curvature, and suggested that multiple ulceration of the stomach might be due to multiple heterotopia. He also drew attention to the fact that repeated intestinal hæmorrhage may be one of the complications of Meckel's diverticulum, and quoted illustrative cases emphasizing the possible diagnostic importance of such hæmorrhage, notably in male children.

Dr. FRASER inquired whether heterotopiæ were found in the appendix.

Mr SCARFF inquired whether the small white lines seen through the œsophagoscope sometimes in cases of dysphagia occurring in women of about forty, might represent heterotopic patches of gastric mucosa.

Dr. TAYLOR, in replying, said he had no certain knowledge of Fallopian diverticula, but considered that they were propably of a post-inflammatory nature. He saw no reason for the belief that adenomyoma of the uterus was of fœtal origin, but regarded it as of an acquired type. He agreed that repeated intestinal hæmorrhage in male children should suggest the possibility of peptic ulceration of a Meckel's diverticulum. He thought diverticula of the appendix were of the nature of pressure diverticula, as their microscopical structure showed that they were protrusions of mucous membrane through a damaged muscular wall. He considered it highly unlikely that the lines seen in the œsophagus were heterotopic in origin, and queried whether an actual ulcer had ever been seen in such cases.

---

The eighth and final meeting of the session was held at the University on 13th May, 1931. The President was in the Chair.

Dr. F. J. A. MAYES showed a number of X-rays, Dr. A. L. TAYLOR pathological specimens, and Mr. BUSH and Dr. SUMMERS an X-ray print of a dermoid cyst.

Dr. H. C. BRISTOWE then read a paper on "**Sterilization of the Unfit,**" which will appear, with the ensuing discussion, in the next issue of the *Journal*.

